# Quality of life analysis after stereotactic radiofrequency ablation of liver tumors

**DOI:** 10.1038/s41598-020-69331-1

**Published:** 2020-07-30

**Authors:** Peter Schullian, Anja Gertl, Gregor Laimer, Daniel Putzer, Uwe Siebert, Elliot Levy, Reto Bale

**Affiliations:** 1 Section of Interventional Oncology-Microinvasive Therapy (SIP), Department of Radiology, Medical University of Innsbruck, Anichstraße 35, 6020 Innsbruck, Austria; 2 Department of Public Health, Health Services Research and Health Technology Assessment, Institute of Public Health, Medical Decision Making and Health Technology Assessment, UMIT-University for Health Sciences, Medical Informatics and Technology, Eduard-Wallnoefer-Zentrum 1, 6060 Hall in Tyrol, Austria; 3 Radiology and Imaging Sciences, National Institutes of Health, Bethesda, MD USA

**Keywords:** Liver cancer, Surgical oncology

## Abstract

The purpose of this study was to evaluate the health-related quality of life (HRQoL) after stereotactic radiofrequency ablation (SRFA) of liver tumors and identify variables associated with decreased HRQoL and to compare it to other treatments in case of concurrency. From 2011 to 2017 577 patients underwent SRFA for liver tumors in 892 ablation sessions. 303 (52.5%) patients completed a HRQoL questionnaire once after the ablation. HRQoL was assessed by the Short Form (SF)-12 health survey with mental and physical component scales and by a general questionnaire to assess disease and treatment-specific items as well as to compare tolerability of SRFA to transarterial chemoembolization (TACE), hepatic resection (HR) and chemotherapy (CTX). The median SF-12 PCS was 46.6 and MCS was 53.2. Patients experiencing pain or complications and patients refusing repeat SRFA showed significantly lower PCS (43.2 vs 48.6, p = 0.0003; 32.8 vs 46.9, p = 0.005 and 40.6 vs 46.9, p = 0.009). 355 (97.8%) patients indicated willingness to undergo repeat SRFA with little to no fear in 292 (80.7%) patients. Among patients with multiple therapies, SRFA was rated by 40 (90.9%) as preferred re-treatment, HR by 1 (2.3%) and CTX by 3 (6.8%). In conclusion, we have shown that SRFA has good HRQoL-outcomes and reported low morbidity rates. Consequently the vast majority of study patients would accept a repeated SRFA if necessary (97.8%), without fear (80.7%). SRFA was preferred among patients who experienced concurrent treatments, such as HR or CTX.

## Introduction

Health-related quality of life (HRQoL) can be defined as “an overall sense of well-being, including aspects of happiness and satisfaction with life as a whole, which is measurable through mental well- being, physical functioning and overall health status”^1^. Recently, the multimodal concept of HRQoL has emerged as an important comparator for available treatments and as a valuable outcome measure distinct from long-term survival and tumor recurrence. Regarding hepatic neoplasms, hepatic resection (HR) is considered to be the gold standard in the management of both primary and metastatic disease. However, HR is still associated with relatively high morbidity rates up to 59.9%^2–5^ and with a corresponding initial decline in HRQoL after surgery^6^. Radiofrequency ablation (RF) affords more rapid post-procedure recovery and thus good post-procedure HRQoL by virtue of its minimally invasive nature, and has been increasingly accepted as an alternative in the management of primary or metastatic liver tumors^7,8^. Supporting this, Huang et al. 2014^9^ reported better post-treatment HRQoL after RFA compared to that after HR in patients with solitary small HCC. Impairment in HRQoL has been reported in patients with hepatocellular carcinomas (HCC)^10,11^, however long-term preservation^12^ after TACE in patients with unresectable HCC and only marginally affection after HR in patients with primary and secondary liver tumors, respectively, have been reported^13^. SRFA includes three-dimensional pre-ablation planning for optimal alignment of multiple RF probes to create larger coagulation volumes, permitting more complex ablations. It’s unknown, to our knowledge, if this increased complexity compared to conventional single probe RF ablation might affect the post-procedure HRQoL. The present study was designed to assess the post-procedure HRQoL after SRFA in patients with primary or secondary liver tumors and to compare it to other concurrent treatments, such as TACE, CTX or HR. Our hypothesis states that, despite the complexity of the stereotactic RF procedure, HRQoL after SRFA is better compared to that after HR or CTX and comparable to that after TACE.

## Materials and methods

### Study population

The local institutional review board approved this study. All patients provided informed consent for both SRFA and QoL assessment. Since 2011, all patients undergoing SRFA for the treatment of primary or secondary liver tumors were asked to complete a HRQoL questionnaire once after the RF procedure (within first few weeks). Until 2017, of a total of 577 treated patients, 303 (52.5%) patients returned 363 questionnaires (311 via mail and 52 via telephone, 50 patients > 1 questionnaires in case of > 1 SRFA sessions). The SF-12 part was correctly completed in 337 of 363 cases (93%). The detailed patient characteristics are presented in Table 1.

**Table 1:** Demographics of the study population with 303 patients.

Characteristics	n
Age, years (range)	64 (9–87)
Sex (female/male), n (%)	90/213 (29.7/70.3)
**Primary liver tumor, n (%)**	159 (52.5)
HCC, n (%)	137 (45.2)
CCC, n (%)	15 (5.0)
Hepatoblastoma, n (%)	1 (0.3)
Benign (FNH, Adenoma), n (%)	6 (2.0)
**Metastatic liver tumor, n (%)**	144 (47.5)
Colorectal, n (%)	79 (26.1)
Neuroendrocrine, n (%)	13 (4.3)
Melanoma, n (%)	8 (2.6)
Mamma, n (%)	8 (2.6)
Other, n (%)	36 (11.8)
Number of tumors per patient, median (range)	1 (1–9)
Size of tumors (cm), median (range)	2.6 (0.6–18)
Number of ablation sessions, median (range)	1 (1–5)
**Previous therapy, n (%)**	162 (53.5)
HR, n (%)	46 (15.2)
CTX, n (%)	75 (24.8)
TACE, n (%)	31 (10.2)
Conventional RFA, n (%)	10 (3.3)
**Liver cirrhosis, n (%)**	118 (38.9)
Child A, n (%)	100 (84.7)
Child B, n (%)	17 (14.4)
Child C, n (%)	1 (0.8)

*HCC* hepatocellular carcinoma, *CCC* cholangiocellular carcinoma, *HR* hepatic resection, *CTX *chemotherapy, *TACE *transarterial chemoembolization, *FNH *focal nodular hyperplasia, *RFA* radiofrequency ablation.

**Figure 1:**
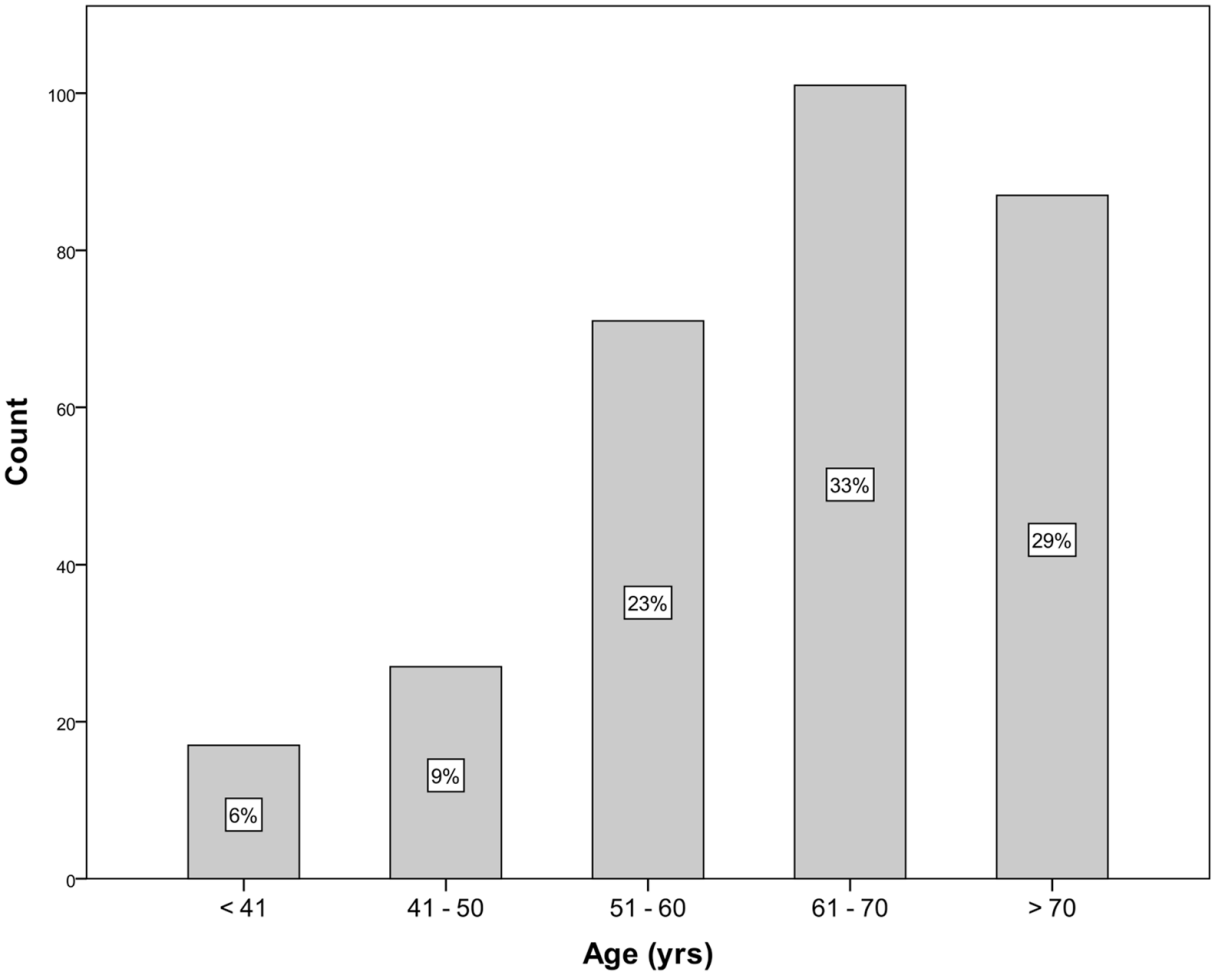
Age distribution in study population.

**Table 2:** SF-12 results in comparison to normal population.

	Score	Study population			Normal population		
		n	Median	IQR	n	Median	IQR
Total	PCS	337	46.6	39.1–53.5	2,805	52.8	44.2–54.8
	MCS	337	53.2	47.1–57.9	2,805	54.2	48.3–57.8
Male	PCS	244	46.9	40.8–54.0	1,254	53.5	46.7–55.0
	MCS	244	53.7	47.8–58.0	1,254	55.2	50.6–57.9
Female	PCS	93	43.5	35.8–52.7	1,551	51.5	42.1–54.6
	MCS	93	52.2	42.1–57.0	1,551	53.2	46.2–57.1

*PCS *physical component summary, *MCS *mental component summary.

**Table 3:** SF-12 results in comparison to normal population according to age.

Age	Score	Study population			Normal population		
		n	Median	IQR	n	Median	IQR
≤ 40	PCS	18	36.8	34.2–50.2	539	54.0	50.8–55.7
	MCS	18	53.6	45.6–60.3	539	53.8	49.5–57.5
41–50	PCS	27	46.9	39.4–55.2	411	53.6	48.0–54.8
	MCS	27	53.7	43.6–58.4	411	54.6	49.5–57.8
51–60	PCS	79	47.1	42.4–54.8	521	50.6	40.3–54.1
	MCS	79	53.4	49.6–57.9	521	53.6	46.0–57.7
61–70	PCS	117	45.9	39.1–53.4	421	46.5	36.3–52.6
	MCS	117	52.9	46.7–57.8	421	55.6	49.7–58.0
> 70	PCS	96	46.4	37.9–52.2	317	40.2	29.4–48.5
	MCS	96	53.3	45.2–57.7	317	53.9	45.8–58.8

*PCS *physical component summary, *MCS *mental component summary.

**Table 4:** Correlation of SF-12 scores according to demographic and clinical factors. Significant p levels (<0.05) are in bold.

	n	PCS Median (IQR)	p	n	MCS Median (IQR)	p
**Total**	337	46.6 (39.1–53.5)		337	53.2 (47.1–57.9)	
**Sex**			0.077			0. 012*
Male	244	46.9 (40.8–54.0)		244	53.5 (47.8–58.0)	
Female	93	43.5 (35.8–52.7)		93	52.2 (42.1–57.0)	
**Age**			0.140			0.644
≤ 40	18	36.8 (34.2–50.2)		18	53.6 (45.6–60.3)	
41–50	27	46.9 (39.4–55.2)		27	53.7 (43.6–58.4)	
51–60	79	47.1 (42.4–54.8)		79	53.4 (49.6–57.9)	
61–70	117	45.9 (39.1–53.4)		117	52.9 (46.7–57.8)	
> 70	96	46.4 (37.9–52.2)		96	53.3 (45.2–57.7)	
**Cirrhosis**			0.807			0.539
Yes	123	46.6 (40.2–52.3)		123	53.4 (47.8–58.3)	
No	211	46.8 (38.9–54.6)		211	53.2 (46.5–57.8)	
**Tumor type**			0.331			0.538
HCC	145	47.0 (41.7–54.2)		45	52.9 (47.1–57.9)	
CCC	25	42.2 (36.8–55.5)		6	54.7 (46.2–57.9)	
Metastases	159	45.9 (38.1–53.4)		58	53.4 (47.1–57.8)	
Benign	7	37.1 (34.3–48.4)		7	51.0 (40.7–60.2)	
**Tumor size**			0.191			0.680
< 3 cm	182	46.9 (41.4–53.4)		182	53.5 (47.1–57.8)	
> 3 cm	151	44.3 (37.7–54.1)		151	52.7 (46.4–58.1)	
**Tumor number**			0.315			0.390
n = 1	182	46.9 (40.7–53.5)		182	53.7 (47.2–58.1)	
n > 1	152	44.3 (38.1–54.5)		152	52.8 (47.0–57.8)	
**Pain after RFA**			0.000*			0.090
Present	180	43.2 (36.2–50.7)		180	52.5 (45.7–57.5)	
Absent	157	48.6 (42.6–55.3)		157	54.0 (48.5–58.1)	
**Complications**			0.005*			0.317
Present	29	40.6 (33.1–47.5)		29	49.6 (40.2–60.1)	
Absent	307	46.9 (40.0–54.6)		307	53.3 (47.2–57.8)	
**Redo RFA**			0.009*			0.011*
Yes	330	46.9 (39.5–53.5)		330	53.3 (47.2–57.9)	
No	7	32.8 (30.2–39.2)		7	42.6 (38.8–50.8)	

*PCS* physical component summary, *MCS *mental component summary, *HCC *hepatocellular carcinoma, *CCC *cholangiocellular carcinoma.

### SRFA procedure

The method of SRFA has previously been reported in detail^14–16^. In brief, the key steps are as follows:

(I) Preparation: treatment is performed under general anesthesia with muscle paralysis and immobilization facilitated by a single (Bluebag, Medical Intelligence Schwabmünchen, Germany) or double vacuum fixation technique (BodyFix, Medical Intelligence, Germany); 10–15 broadly to the skin attached registration markers (Beekley Spots, Beekley Corporation, USA) are used for image-to-patient registration.

(II) Planning: contrast-enhanced (CE) CT (SOMATOM Sensation Open, Siemens AG, Germany) with 3 mm slice thickness in the arterial and portal-venous phase; the obtained CT-data is transmitted to an optical based navigation system (Stealth Station Treon plus, Medtronic Inc., USA); needle trajectories are planned using multiplanar and reconstructed 3D images.

(III) Execution—needle placement: to minimize respiratory motion, temporary disconnections of the endotracheal tube are executed during the planning CT, each stereotactic needle placement and the final CT; after registration, an accuracy check and sterile draping, the ATLAS aiming device (Medical Intelligence Inc., Germany) is used for navigated trajectory alignment; 15G, 17.2 cm coaxial needles (Bard Inc., USA) are placed with the aiming device (without real-time imaging); the coaxial needles serve as guides for the radiofrequency electrodes; to verify proper needle placement, an unenhanced CT is fused with the planning CT by the navigation system; a 16G biopsy sample is obtained via one of the coaxial needles in patients with lack of histological confirmation.

(III) Execution—RF ablation: three 17G radiofrequency electrodes (Cool-tip, Medtronic, USA, 25 cm length with 3 cm exposure) are simultaneously placed through the coaxial needles for serial ablation using the unipolar Cool-tip radiofrequency generator with switching control (Cool-tip, Medtronic, USA); the standardized ablation time for three electrodes is 16 min; however, in case of significant increase of impedance (the so-called roll-off effect) the ablation process is finished; track ablation is done during every repositioning and final removal to avoid bleeding and tumor seeding;

(IV) Final control: post-ablation completion contrast-enhanced CT in the arterial and portal venous phase, fused with the planning CT for verification of ablation zone coverage and for assessment of complications;

### Comparative treatments

In CTX, therapeutics were used in standard dosage, frequency and duration according to international guidelines. All liver resections were performed under laparotomy. TACE was carried out in standard technique with administration of an emulsion of Lipiodol (Guerbet, Roissy, France) with epi- or doxorubicine. None of the patient was treated by stereotactic body radiotherapy for liver tumors.

### Instruments for HRQoL assessment

The HRQoL was primarily investigated using a specifically developed questionnaire consisting of general items, disease and current therapy related items, symptoms after treatment related items and if available, other items with direct comparison of previous and or additional treatments, such as CTX, TACE or HR (see Table 5 for details).

**Table 5:** Details of the questionnaires.

Section	Item	
I. General items	Sex	Male/female
	Age	Years
	Retired	Yes/no
II. Disease specific items	Liver tumor	Primary
	Comorbidities	Secondary
III. Therapy related items	Medical consultant	
	Current therapy	
	Regular medical follow-up	Yes/no
	Redo SRFA	Yes/no
	Fear of repeat SRFA	Yes/no
	Physical state after SRFA	Better/worse/equal
IV. Symptoms after SRFA	Pain	Scale, duration
	Fever	Yes/no, duration
	Poor wound healing	Yes/no
	Digestive disturbances	Yes/no, duration
	Reflux	Yes/no, duration
	Night sweats	Yes/no, duration
	Nausea/vomiting	Yes/no, duration
	Lack of appetite	Yes/no, duration
	Weakness	Yes/no, duration
V. Other treatments (not SRFA) with comparison	Treatment	TACE, CTX, HR
	Direct comparison of treatments	Side-effects
		Physical stress
		Impairment
		Preference
VI. Suggestions for Improvement	–	–
VII. SF-12	Physical health related	General health (GH)
		Physical functioning (PF)
		Role physical (RP)
		Body pain (BP)
	Mental health related	Vitality (VT) Social functioning (SF) Role emotional (RE) Mental health (MH)

For comparison and reproducibility an internationally validated questionnaire, SF 12 consisting of twelve questions that measure eight health domains to assess physical and mental health, was used. Physical and mental component summary (PCS and MCS) are computed using the scores of twelve questions and range from 0–100, where 0 indicates the lowest level of health and 100 indicates the highest level of health. We administered the questionnaire once after treatment within the first few weeks. The comparative values of the German normal population were gathered from the SF-12 scoring manual.

### Statistical analysis

SPSS (SPSS Inc., Chicago, USA; version 24.0) was used for statistical analysis. Continuous variables were presented as the median with range. Categorical variables were reported both as numbers and as percentages. The Wilcoxon signed rank test was used for 1-sample testing. The distributions of categorical and numerical variables between independent groups were compared using Fisher’s exact test, Mann–Whitney *U* test (two independent variables) and Kruskal–Wallis test (more than two independent variables), respectively. A *p* value < 0.05 was considered as statistically significant.

## Results

### Patient characteristics (Table 1)

Between 2011 and 2017, 577 patients underwent SRFA for primary and secondary liver tumors in 892 RFA sessions. 303 (52.5%) returned 363 questionnaires (311 via mail, 52 via telephone). The SF-12 part was correctly completed in 337 of 363 cases (93%). 213 (70.3%) were male and 90 (29.7%) female. The median age was 64 years (range 9–87). Figure 1 shows the corresponding age-distribution. 159 (52%) patients had primary and 144 (47.5%) metastatic liver tumors, with 79 (26.1%) from colorectal cancer, 13 (4.3%) from neuroendocrine tumor, 8 (2.6%) from melanoma and 8 (2.6%) from breast cancer. 118 (38.9%) had liver cirrhosis. At initial therapy the median size of liver tumors was 2.6 cm (range 0.6–18 cm) and the median number of lesions was 1 (range 1–9). 162 (53.5%) patients had previous therapies before initial SRFA, 10 conventional RFA, 31 TACE, 75 CTX and 46 h.

The total number of treated tumors in the study population was 1,092. 87 of 1,092 (8%) tumors showed insufficient local control during follow-up by developing local recurrence.

### SF-12 results (Tables 2, 3, 4; Fig. 2) 

The median PCS was 46.6 (range 39.1–53.3) and MCS 53.2 (range 47.1–57.9). The PCS of patients younger than 40 years in the study group was lower compared to the control population (Fig. 3).

**Figure 2:**
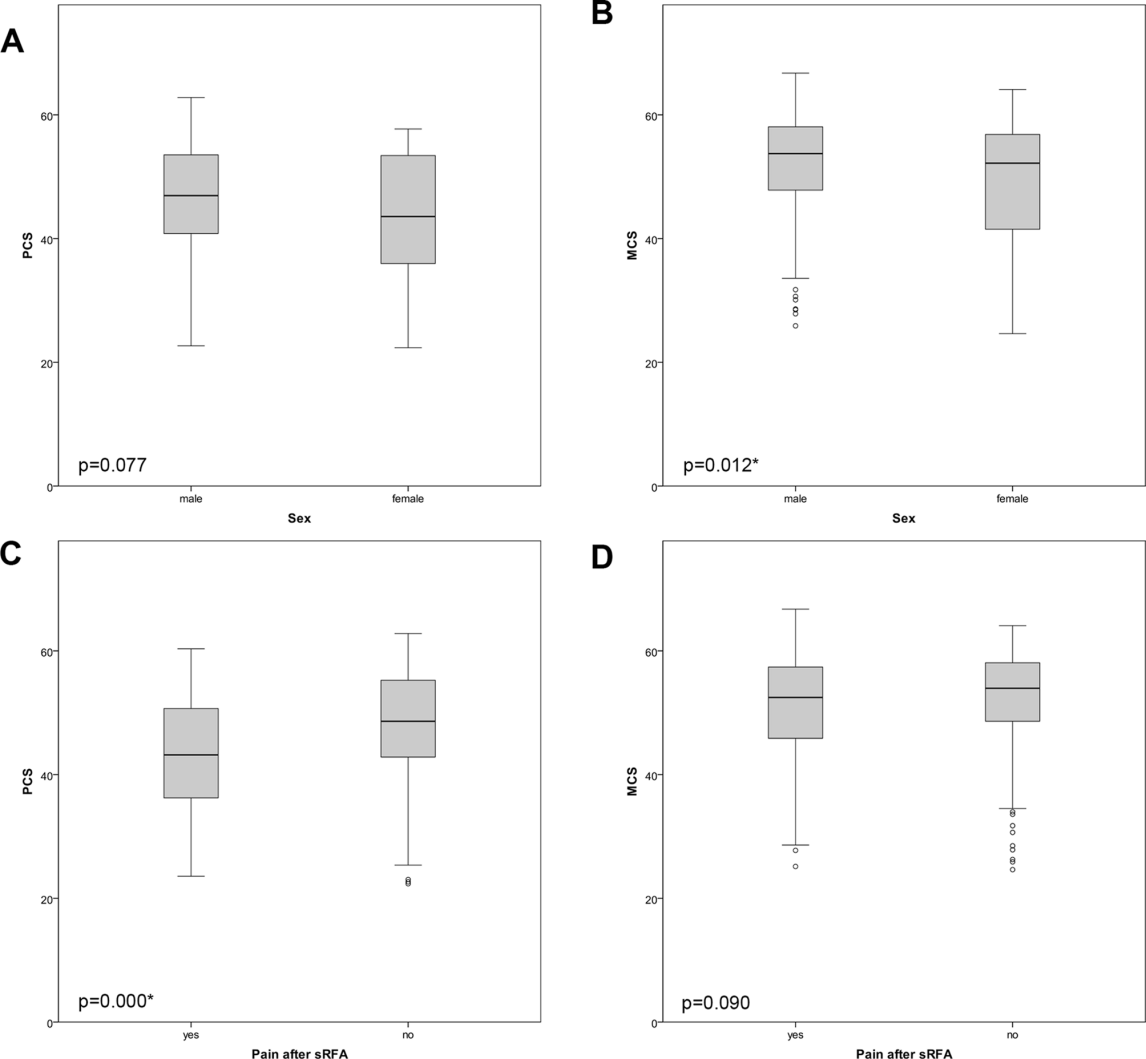

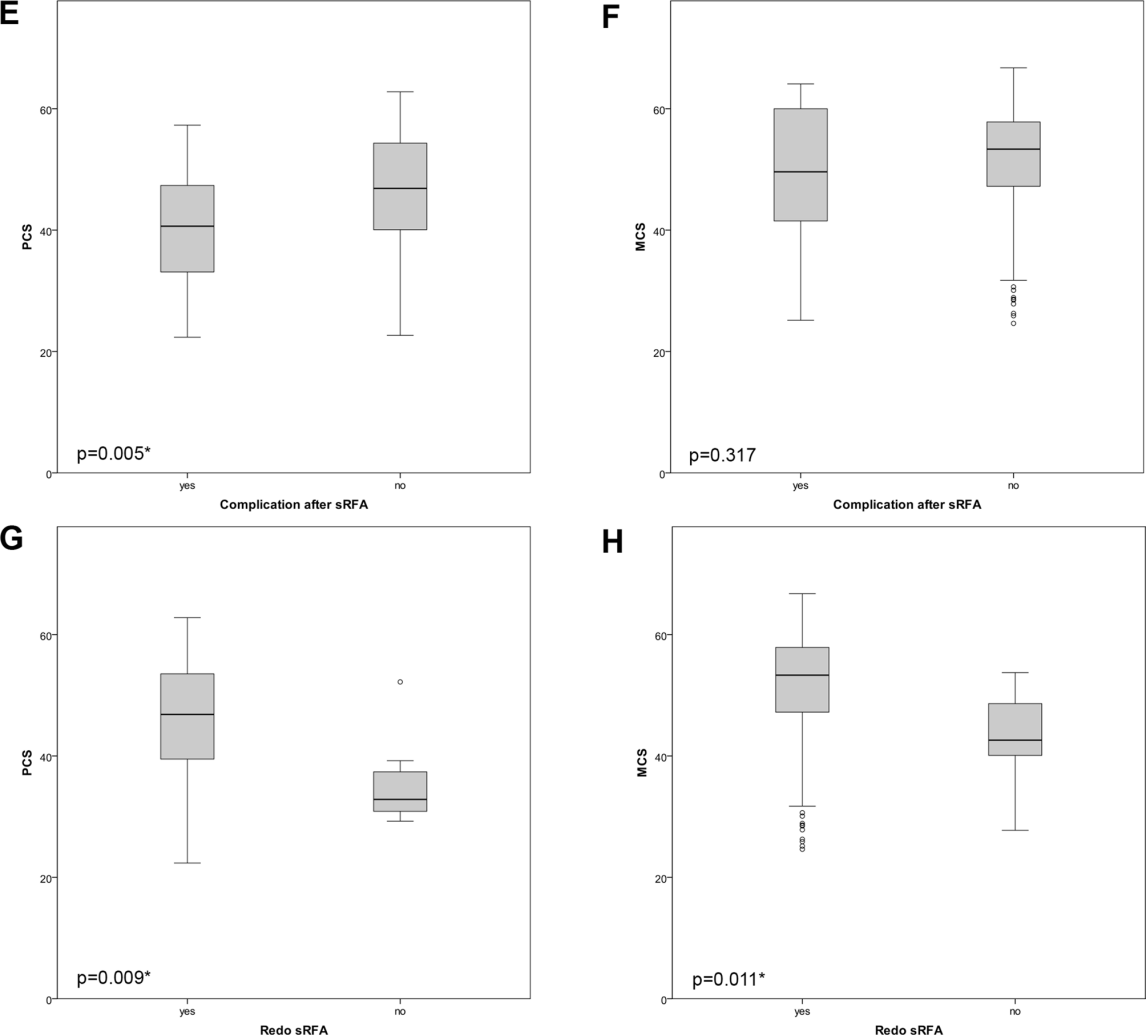
Boxplots of PCS (**A**, **C**, **E**, **G**) and MCS (**B**, **D**, **F**, **H**) according to different factors.

**Figure 3:**
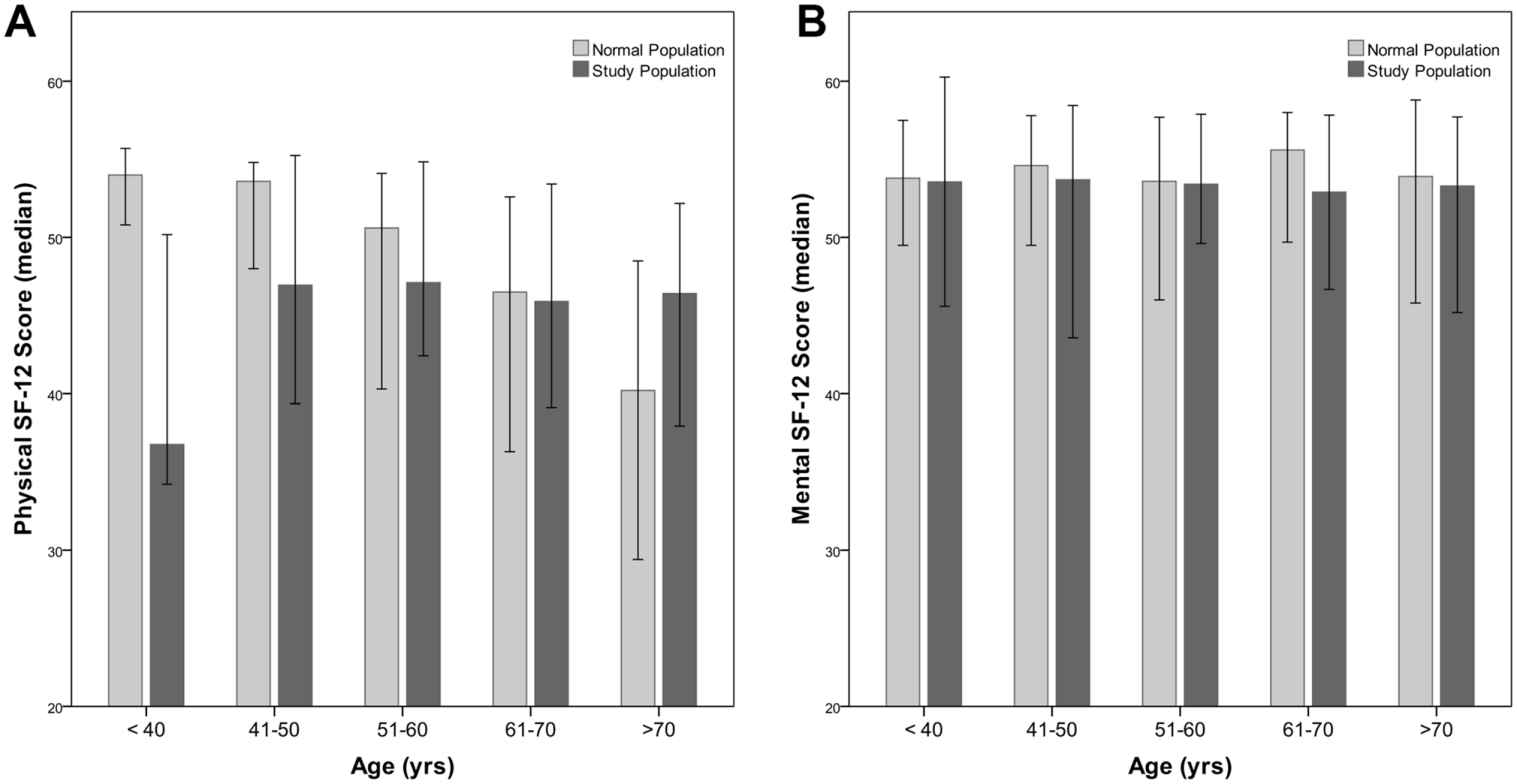
Showing physical and mental scores compared to normal population.

Age, liver cirrhosis or tumor type, number and size did not significantly influence the SF-12 scores. Patients refusing repeated SRFA had a significantly lower PCS and MCS with p = 0.009 and p = 0.011, respectively. Compared to males, females had significantly lower MCS, with p = 0.012. Patients experiencing pain or complications after SRFA showed significantly lower PCS with p = 0.000 and p = 0.004, respectively.

### Study questionnaire (Table 5)

#### Subjective HRQoL and symptoms after SRFA (Fig. 4)

**Figure 4:**
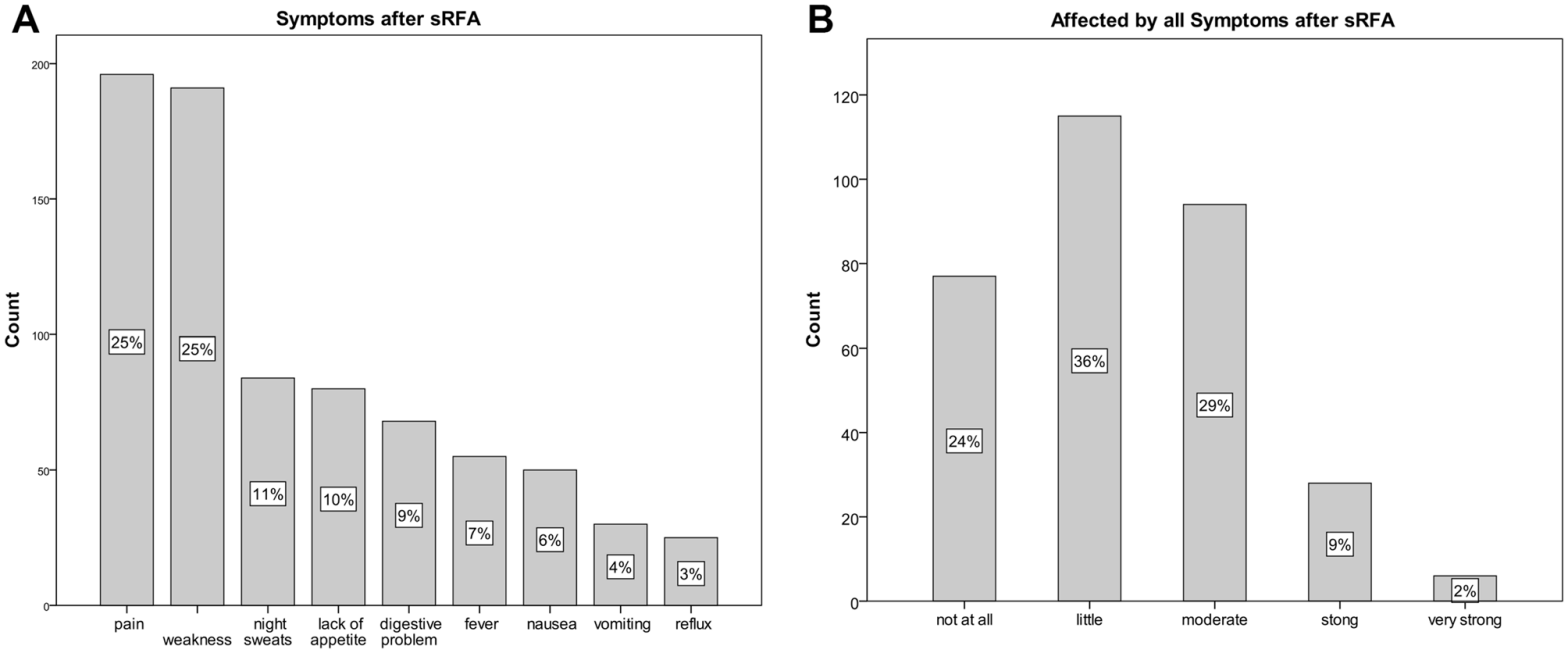
Showing symptoms (**A**) and its affection (**B**) after SRFA.

248/363 (68.3%) patients rated their physical state as very good or good after intervention, 89 (24.5%) as satisfying and 22 (6.1%) as insufficient, respectively. 346/363 (95%) rated their health condition after intervention as equal to or better than before SRFA. On average, patients stated that it took a few days to a few weeks to be as fit as before the intervention. 195/363 (53.7%) patients had pain after the intervention, 42/195 (21.5%) higher than VAS 6, which lasted for a few days to a few weeks. 55/363 (15.2%) suffered from fever, 32/363 (8.8%) from wound healing deficiencies, 68/363 (18.7%) from digestive disorders, 25/363 (6.9%) from acid reflux, 80/363 (22%) from lack of appetite, 50/363 (13.8%) from nausea, 30/363 (8.3%) from vomiting, 84/363 (23.1%) from night sweat, 191/363 (52.6%) from feeling weak. None of the indicated symptoms lasted longer than a few weeks. 192/363 (60%) patients were in no sense or minimally, and 34 (10.7%) strongly affected by the above-mentioned symptoms.

#### Treatment burdening (Fig. 5)

**Figure 5:**
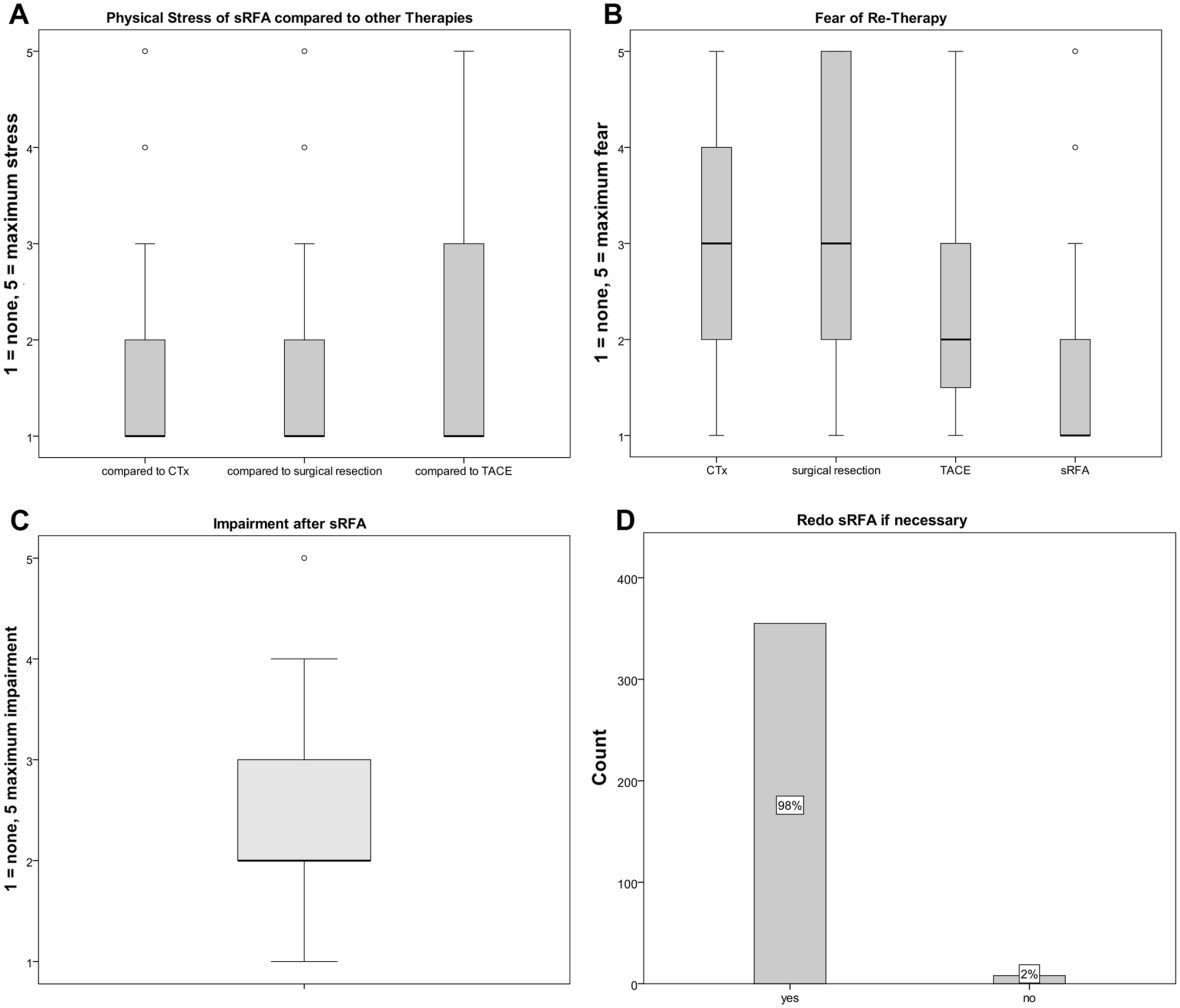
Physical stress of SRFA compared to other therapies (A), fear of re-treatment (B), physical impairment after SRFA (C), fear of re-SRFA (D).

Patients experiencing multiple therapies (n = 147) were asked to arrange treatments according to their burden: CTX and HR where rated as most burdening by 68/114 (59.6%) and 32/62 (51.6%), SRFA by 9/147 (6.6%) and TACE by 2/31 (6.5%). Patients rated SRFA as non/little-burdening and moderate-burdening in 86/147 (58.7%) and 47/147 (32.3%) cases, respectively, with a significantly lower physical stress after SRFA compared to other therapies (p = 0.000, Fig. 5A). CTX was considered as the therapy with most side-effects (84/147 patients, 57.1%), followed by resection (50/147 patients, 34.1%), SRFA (11/147 patients, 7.5%) and TACE (2/147 patients, 1.4%). From the entire study population, 192/363 (60%) patients were none or minimally affected by SRFA. 13/363 (3.6%) had to be medically treated after intervention. The median in-patient stay was 3 days (range 1–21 days) after SRFA, 2.5 days (range 1–60 days) after TACE and 14 days (range 4–100 days) after HR (significantly higher compared to SRFA and TACE with p = 0.000 and p = 0.001, respectively). The median sick leave after SRFA was 7 days (range 1–90 days).

#### Fear of re-therapy (Fig. 5B)

If necessary, 355/363 (97.8%) patients indicated willingness to undergo repeat SRFA, with 292/363 (80.7%) patients having little to no fear. In comparison, 18/363 (28.1%) patients would have no or very little fear of repeated HR, 7/363 (58.3%) of TACE and 35/363 (31.5%) of CTX, respectively. Among patients with multiple therapies, SRFA was rated by 132/147 (89.8%) as the preferred re-treatment, HR by 4/147 (2.7%) and CTX by 11/147 (7.5%).

### Ethical approval

All procedures performed in studies involving human participants were in accordance with the ethical standards of the institutional and/or national research committee and with the 1964 Helsinki declaration and its later amendments or comparable ethical standards.

### Informed consent

Informed consent was obtained from all individual participants included in the study.

### Consent for publication

Consent for publication was obtained for every individual person’s data included in the study.

## Discussion

We have shown that SRFA has good HRQoL outcomes in a large series of patients and is perceived as less burdensome compared to HR or CTX to those patients who experienced both treatments. HR is still considered as the preferred treatment for primary and secondary liver tumors despite significant physical and psychological burdens. Recently minimally invasive curative treatments have gained more importance due to lower morbidity rates paired with comparable oncological outcomes^17–23^. SRFA has shown to be as effective as HR in the treatment of colorectal and breast cancer liver metastases, HCC and ICC^15,24–26^. HRQoL has become increasingly important as an outcome measure and has not yet been investigated for SRFA treatments. The present study evaluated HRQoL after SRFA in patients with primary and secondary liver tumors using two different instruments.

In this study, patients older than 40 years showed comparable overall PCS after the RF procedure compared to that of the general German control population. This is in contrast to HRQoL reports after HR, where HRQoL regularly shows an initial decline with considerable improvement after 6 months with the highest scores after 24 months^6^. According to Toro et al. and Dasgupta et al.^27,28^ this pattern may be indicative of the regenerative capacity of the liver. Liver disease, age, tumor type, number and size did not significantly influence PCS or MCS in the present study. In agreement with the study of Poon et al. and Wang et al. 2007^29,30^, pain after the procedure had a significantly negative impact on the PCS (43.2 vs 48.6, p = 0.0003). Patients refusing repeated SRFA had significantly lower PCS and MCS (32.8 vs 46.9, p = 0.009; 42.6 vs. 53.3, p = 0.011, respectively). In accordance, patients experiencing complications after SRFA showed significant lower PCS with 40.6 vs 46.9 (p = 0.005), whereas MCS was not significantly different with 49.6 vs 53.3 (p = 0.317), respectively.

In patients with small HCC, Huang et al. 2014^9^ reported better HRQoL after percutaneous RFA than after HR^30,31^, however with marked improvement 3–6 months after therapy in HR patients. The worsened HRQoL is explained by direct consequences of surgery, especially in the early postoperative period. In agreement, He et al. 2018^32^ reported improved or stable HRQoL after RF ablation in HCC patients. In the present study, SRFA was preferred to CTX or HR by patients that experienced both treatments indicated by significantly lower (p = 0.000, Fig. 5A) median rated scores of physical stress. Given the increased complexity of SRFA compared to conventional RFA, these results may be surprising, but they underline that the complexity of SRFA does not hinder rapid post-procedural recovery. In line with our hypothesis, TACE showed comparable results in HRQoL. However, since conventional US- and CT-guided “freehand” RFA was completely replaced by SRFA at our intuition in 2002, we could not show any direct comparisons to conventional RFA. The most common reported symptoms such as pain or weakness (54% and 53%) were only temporary (up to a few weeks) and their rated impairment was low (median 2, Fig. 5B). This compares favorably with the reports after HR, where pain, dyspnoea and fatigue remained even at 36–48 months^28^. Toro et al. 2012^27^ reported that patients who were treated by TACE showed a significant long term reduction of physical well-being, social/family well-being, emotional well-being, functional well-being and additional concerns. The potential explanation is that patients who undergo TACE commonly have already developed advanced liver or cancer disease, respectively.

SRFA in its current from was introduced in 2003. A few technical improvements and modifications in the procedure were introduced thereafter. However, during the study period from 2011–2017 sRFA technology and procedure did not change anymore.

The complication rate reported by the patients was higher than that of the institutional medical reports (31, 8.6% vs. 16, 4.4%). Patients might have overrated complications as major complications or patients experiencing complications might be more likely to reply to the questionnaire than asymptomatic patients (selection bias). The presence of complications was associated with a significant decline in PCS (40.6 vs 46.9, p = 0.004).

The median reported in-patient-stay was significantly lower (3 days vs. 14 days, p = 0.000) after SRFA compared with that after HR, however not significantly different compared to that after TACE (3 days vs. 2.5 days, p = 1.000).

## Limitations

The limitations of the study are related to its single-arm design with heterogeneous patient collectives—some patients underwent only SRFA, while others received SRFA together with different additional therapies. Compared to other studies, HRQoL in the present study was obtained only once after therapy and thus no long-term HRQoL predictions could be made. The return rate of the questionnaire was 52.5%, with a relative major complication rate of 4.4% in the study group vs. 7.4% in the non-responder group. Hence, a possible bias could be that some patients who have suffered complications did not return a questionnaire. In addition, the cultural diversity of the study population could also represent a possible bias in the subjective assessment of quality of life.

## Conclusion

We have shown that SRFA has good HRQoL-outcome. The vast majority of patients in our survey would accept a repeated SRFA if necessary (97.8%), without fear (80.7%) and is preferred among patients who experienced concurrent treatments, such as HR or CTX.

## Abbreviations

CTX: Chemotherapy
HCC: Hepatocellular carcinoma
HR: Hepatic resection
ICC: Intrahepatic cholangiocarcinoma
MCS: Mental component summary
PCS: Physical component summary
HRQoL: Health-related quality of life
RF: Radiofrequency
SRFA: Stereotactic radiofrequency ablation
TACE: Transcatheter arterial chemoembolization
